# Quantitative magnetic resonance image assessment of the optic nerve and surrounding sheath after spaceflight

**DOI:** 10.1038/s41526-020-00119-3

**Published:** 2020-10-08

**Authors:** Jesse J. Rohr, Stuart Sater, Austin M. Sass, Karina Marshall-Goebel, Robert J. Ploutz-Snyder, C. Ross Ethier, Michael B. Stenger, Bryn A. Martin, Brandon R. Macias

**Affiliations:** 1 Neurophysiological Imaging and Modeling Laboratory, University of Idaho, 875 Perimeter Drive MC1122, Moscow, ID 83844-1122 USA; 2 KBR, Houston, TX USA; 3 Applied Biostatistics Laboratory, School of Nursing, University of Michigan, Ann Arbor, MI USA; 4 Wallace H. Coulter Department of Biomedical Engineering, Georgia Institute of Technology and Emory University, Atlanta, GA USA; 5 Cardiovascular and Vision Laboratory, Johnson Space Center, National Aeronautics and Space Administration, Houston, TX USA

**Keywords:** Vision disorders, Neuroscience, Optic nerve diseases, Biomedical engineering

## Abstract

A subset of long-duration spaceflight astronauts have experienced ophthalmic abnormalities, collectively termed spaceflight-associated neuro-ocular syndrome (SANS). Little is understood about the pathophysiology of SANS; however, microgravity-induced alterations in intracranial pressure (ICP) due to headward fluid shifts is the primary hypothesized contributor. In particular, potential changes in optic nerve (ON) tortuosity and ON sheath (ONS) distension may indicate altered cerebrospinal fluid dynamics during weightlessness. The present longitudinal study aims to provide a quantitative analysis of ON and ONS cross-sectional areas, and ON deviation, an indication of tortuosity, before and after spaceflight. Ten astronauts undergoing ~6-month missions on the International Space Station (ISS) underwent high-resolution magnetic resonance imaging (MRI) preflight and at five recovery time points extending to 1 year after return from the ISS. The mean changes in ON deviation, ON cross-sectional area, and ONS cross-sectional area immediately post flight were −0.14 mm (95% CI: −0.36 to 0.08, Bonferroni-adjusted *P* = 1.00), 0.13 mm^2^ (95% CI −0.66 to 0.91, Bonferroni-adjusted *P* = 1.00), and −0.22 mm^2^ (95% CI: −1.78 to 1.34, Bonferroni-adjusted *P* = 1.00), respectively, and remained consistent during the recovery period. Terrestrially, ONS distension is associated with increased ICP; therefore, these results suggest that, on average, ICP was not pathologically elevated immediately after spaceflight. However, a subject diagnosed with optic disc edema (Frisen Grade 1, right eye) displayed increased ONS area post flight, although this increase is relatively small compared to clinical populations with increased ICP. Advanced quantitative MRI-based assessment of the ON and ONS could help our understanding of SANS and the role of ICP.

## Introduction

**Fig. 1:**
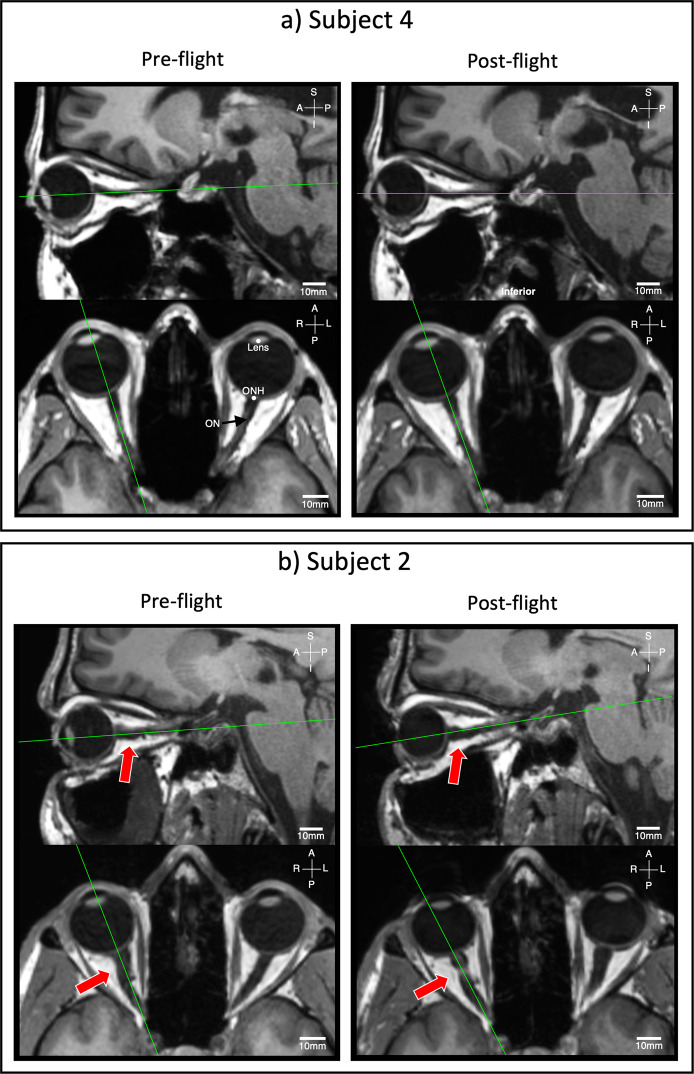
Sample MRIs used for ON deviation quantification. Preflight and R+1 T1-weighted sagittal MRI of 2 astronauts showing astronauts **a** without and **b** with ON deviation after spaceflight in both sagittal (top row) and axial (bottom row) views. Red arrows highlight the largest ON deviation in our cohort and green lines identify the locations of the orthogonal images. S–I superior and inferior, A–P anterior and posterior, R–L right and left.

As a result of extended spaceflight, some astronauts experience ophthalmic changes collectively referred to as spaceflight-associated neuro-ocular syndrome (SANS)^1,2^. The severity of SANS may increase with spaceflight duration and possibly result in permanent ocular structural and functional changes^2^. SANS is characterized by decreased near-visual acuity, globe flattening, optic disc edema, and choroidal folds^3–6^. These changes may be impacted by optic nerve sheath (ONS) distension and optic nerve (ON) tortuosity (Fig. 1) clinically observed in astronauts by Mader et al.^3^ and Kramer et al.^6^ also measured the diameter of the mid-orbit ONS, noting that diameters were significantly larger when kinks were present^6^. These descriptive findings were important but also somewhat limited, with a modest number of quantitative measures obtained in a two-dimensional (2D) magnetic resonance imaging (MRI) plane.

Although the underlying pathophysiology of SANS has not been unequivocally determined, the leading hypothesis is that the absence of gravity and thus hydrostatic pressure gradients acting on the body result in a headward fluid shift and possibly mild, chronic elevation in intracranial pressure (ICP) relative to the upright position on Earth^7^. Cerebrospinal fluid (CSF) pressure is likely equalized across the CSF column in space, resulting in increased CSF pressure within the bulbar subarachnoid space surrounding the ON compared to the upright position on Earth. Therefore, decreased intracranial and ONS compliance may also impose abnormal strains on ON head structures^8–11^. Terrestrially, alterations to intracranial compliance have been implicated in several central nervous system pathologies such as normal-pressure hydrocephalus^12^.

Several terrestrial pathologies have clinical signs and symptoms similar to those seen in astronauts with SANS. For example, ON tortuosity and “kinking,” as well as distension of the ONS, are commonly reported in neurofibromatosis type 1 and idiopathic intracranial hypertension (IIH, also known as pseudotumor cerebri) patients^13–15^. IIH can result in papilledema and periorbital subarachnoid space enlargement, similar to that reported in SANS, and can ultimately lead to permanent visual alterations^16–18^.

A significant knowledge and methodological gap exists in quantification of ocular structural adaptions after spaceflight due to the following: (a) a limited astronaut study population, (b) 2D operator-dependent measurement methods, (c) descriptive measurements often collected at a single time-point post flight, and (d) lack of measurement reliability quantification. Development of automated methods for measuring ONS geometries in three dimension (3D) would thus be of great benefit in quantifying ON-associated changes in SANS. In this study, we developed semi-automated methods to quantify ON deviation as a measure of tortuosity and the bulbar subarachnoid space 3D geometry in astronauts before and after long-duration spaceflight missions. A deeper understanding of how such changes relate to ocular dysfunction in SANS will potentially allow identification of SANS risk factors and countermeasures.

## Results

### Flight duration and demographics

Noninvasive MRI data were collected in ten astronauts preflight (508 ± 230 days before launch) and at five recovery time points after return (R+) to Earth: R + 1 (4 ± 2 days), R + 30 (31 ± 5 days), R + 90 (101 ± 16 days), R + 180 (188 ± 15 days), and R + 360 (355 ± 14 days). The means ± SDs of height, weight, and flight duration for this cohort were 1.8 ± 0.1 m, 76 ± 9 kg, and 167 ± 17 days, respectively. Only one subject in our study cohort was diagnosed with grade 1 optic disc edema (i.e., SANS) via fundus imaging (i.e., SANS).

### Optic nerve deviation

**Fig. 2:**
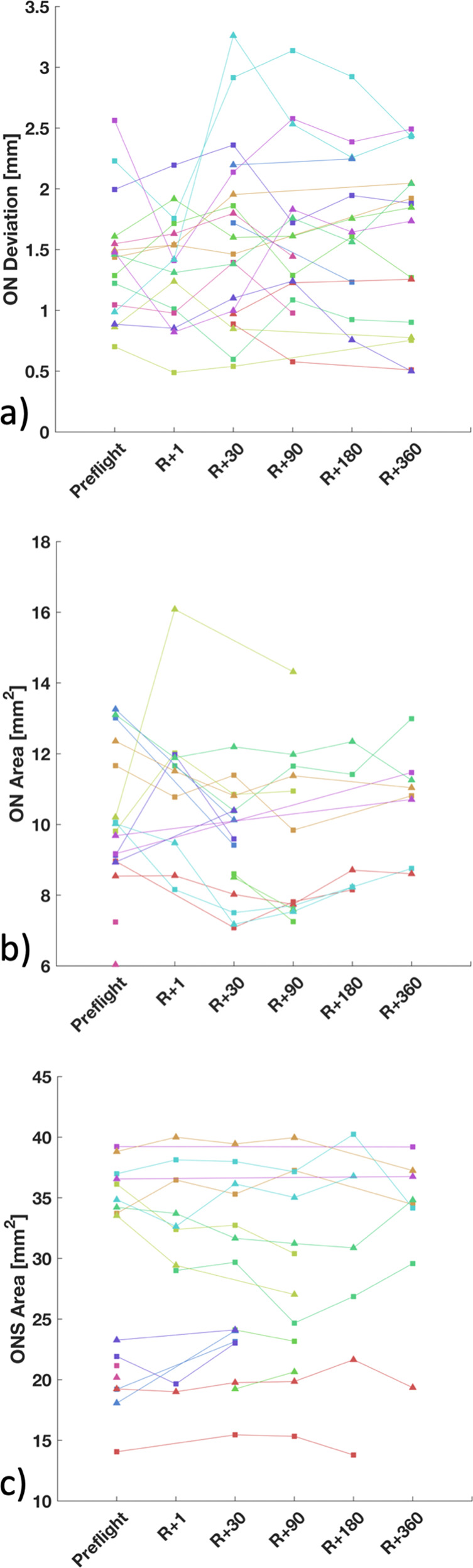
ON deviation, ON cross-sectional area, and ONS cross-sectional area preflight and at multiple time points post flight. Astronaut-specific ON and ONS values measured at different time points (Preflight, R + 1, R + 30, R + 90, R + 180, and R + 360) demonstrating **a** ON deviation, **b** ON cross-sectional area 3 mm posterior to the optic nerve head, and **c** ONS cross-sectional area 3 mm posterior to the optic nerve. Line colors represent individual subjects.

ON deviation from a straight-line path was quantified using a manual image post-processing method to identify potential ON kinking that may have developed during spaceflight (Table 1). Of the ten astronauts analyzed (*n* = 16 eyes, assessed at multiple time points), mean change in ON deviation, adjusting for astronauts’ preflight microgravity exposure, was −0.14 mm (95% confidence interval (95% CI): −0.36 to 0.08 mm, Bonferroni-adjusted *P* = 1.00) (Table 2). Prior comparisons of deviation at each postlanding time point to preflight values showed little change across most time points (Fig. 2a). Examining the individual astronaut plots, there appeared to be two individuals with sizeable increases in deviation between the first two postflight time points (R + 1, R + 30). The astronaut diagnosed with unilateral optic disc edema showed no change in ON deviation immediately post flight (0.04 mm) in the affected eye.

**Table 1:** Marginal means of ON deviation, ON cross-sectional area, ON diameter, ONS cross-sectional area, and ONS diameter measured preflight and at five postflight time points with 95% confidence intervals.

Parameter	Preflight	R + 1	R + 30	R + 90	R + 180	R + 360
ON deviation (mm)	1.41 (1.10 to 1.72)	1.27 (0.95 to 1.59)	1.48 (1.18 to 1.79)	1.66 (1.34 to 1.97)	1.51 (1.19 to 1.82)	1.54 (1.23 to 1.85)
ON cross-sectional area (mm^2^)	9.98 (8.83 to 11.12)	10.11 (8.89 to 11.32)	9.09 (7.94 to 10.25)	9.11 (7.91 to 10.30)	9.61 (8.31 to 10.92)	10.04 (8.79 to 11.29)
ON diameter (mm)	3.56 (3.35 to 3.76)	3.59 (3.36 to 3.80)	3.40 (3.18 to 3.61)	3.41 (3.17 to 3.62)	3.50 (3.25 to 3.73)	3.57 (3.35 to 3.79)
ONS cross-sectional area (mm^2^)	28.08 (22.91 to 33.26)	27.87 (22.63 to 33.10)	28.73 (23.55 to 33.92)	27.38 (22.17 to 32.60)	28.39 (23.05 to 33.73)	27.72 (22.43 to 33.00)
ONS diameter (mm)	5.98 (5.40 to 6.51)	5.96 (5.36 to 6.49)	6.04 (5.48 to 6.57)	5.90 (5.31 to 6.44)	6.01 (5.42 to 6.55)	5.94 (5.34 to 6.48)

**Table 2:** ON deviation, ON cross-sectional area, and ONS cross-sectional area did not change significantly after long-duration spaceflight for the cohort of astronauts analyzed in this study.

Parameter	Average change at R + 1 (95% CI)	*P*-value
ON deviation (mm)	−0.14 (−0.36 to 0.08)	1.00
ON cross-sectional area (mm^2^)	0.13 (−0.66 to 0.91)	1.00
ONS cross-sectional area (mm^2^)	−0.22 (−1.78 to 1.34)	1.00

Key results are reported as the average pre- to postflight change in each parameter with 95% confidence interval (CI) and corresponding Bonferroni-adjusted *P*-values.

### ON cross-sectional area

**Fig. 3:**
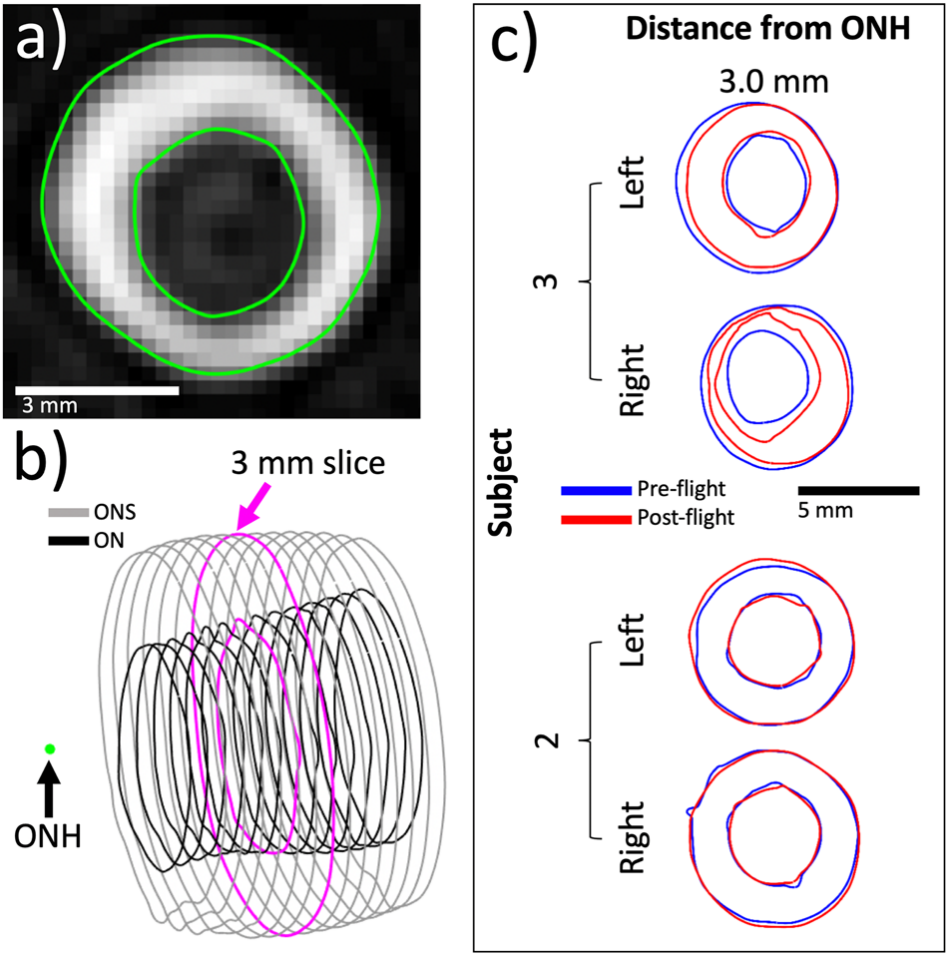
3D ON and ONS assessment methods. **a** Example of the ON (inner green circle) and ONS (outer green circle) boundary contours generated for a slice 3 mm posterior to the optic nerve head. **b** Combined ON (black) and ONS (gray) contours for multiple slices including 3 mm slice contours (magenta), and **c** example pre- vs. postflight ON and ONS contours for two subjects showing the largest ON cross-sectional area increase (top) and largest ONS cross-sectional area increase (bottom) at the 3 mm slice location.

The ON cross-sectional area was measured using semi-automated methods 3 mm posterior to the ONH (Table 1). Adjusting for astronauts’ preflight microgravity exposure, the average change in ON area from pre- to immediate post flight was 0.13 mm^2^ (95% CI: −0.66 to 0.91 mm^2^, Bonferroni-adjusted *P* = 1.00) (Table 2). Only the R + 30 postflight time point showed a statistical difference from preflight, with an average reduction in ON area of −0.89 mm^2^ (95% CI: −1.76 to −0.02 mm^2^, Bonferroni-adjusted *P* = 0.04). On average, ON area tended to decrease from preflight to R + 1, excluding a large increase for one subject (Fig. 2b). Contours for the subject with the largest ON area increase are visualized in Fig. 3c. The single subject diagnosed with grade 1 optic disc edema in our study had an ON cross-sectional area reduction of −0.85 mm^2^ at R + 1 in that eye, a value within the 95% CI for that measurement.

### ONS cross-sectional area

Similar to the ON, ONS cross-sectional area was measured 3 mm posterior to the ONH. Pre- vs. postflight ONS cross-sectional area average change, adjusting for prior microgravity exposure, was −0.22 mm^2^ (95% CI: −1.78 to 1.34 mm^2^, Bonferroni-adjusted *P* = 1.00) and all subsequent comparisons of postflight observations to preflight levels were nonsignificant (Fig. 2c). Correlation between ONS cross-sectional area and deviation was limited, with an *R*^2^ of 0.22. Notably, the single subject in our cohort diagnosed with unilateral grade 1 optic disc edema also displayed the largest increases in ONS cross-sectional area, with an increase of +1.19 mm^2^ in the eye diagnosed with optic disc edema and +2.76 mm^2^ in the other eye (not diagnosed with optic disc edema; however, displayed signs of retinal thickening via optical coherence tomography (OCT)) immediately post flight (R + 1).

### Reliability assessment

Intra- and interscan reliability of results were found to be strong for the automated ON and ONS cross-sectional areas, with a minimum intraclass correlation coefficient (ICC) of 0.71. Intra- and inter-operator reliability for manually selected ON deviation was good (Table 3) with a minimum ICC of 0.67.

**Table 3:** Parameter reliability was quantified as mean ± SD, intraclass correlation coefficient (ICC), and coefficient of variation (CV) based on inter-operator, intra-operator, or phantom model testing.

Parameter	Mean ± SD	%	ICC	CV	Test
ON deviation (mm)	0.15 ± 0.11	11.9 ± 8.8	0.68	NA	Intra-operator
	0.13 ± 0.11	9.9 ± 8.5	0.67	NA	Inter-operator
ON cross-sectional area (mm^2^)	0.36 ± 0.24	1.6 ± 1.1	0.71	0.14	Idealized phantom
	0.23 ± 0.19	1.9 ± 1.4	0.96	0.02	Subject-specific phantom
ONS cross-sectional area (mm^2^)	0.50 ± 0.41	0.9 ± 0.7	0.94	0.09	Idealized phantom
	0.65 ± 0.73	1.0 ± 0.7	0.99	0.01	Subject-specific phantom

For estimated parameter reliability calculations, mean ± SD and corresponding percentage (%) were computed based on mean difference of the individual cases from the average value ± SD of the mean differences (see “Methods”).

## Discussion

Semi-automated MRI-based quantification of intraorbital anatomy revealed that spaceflight did not result in changes in ONS anatomy for the majority of individuals after long-duration spaceflight in our astronaut cohort. In addition, recovery scans collected up to 1 year after spaceflight showed consistency in ONS cross-sectional area measurements. Interestingly, individual ONS cross-sectional areas tended to segment into two populations (Fig. 2b). In our limited study cohort, it is unclear whether this division has any significance. Notably, one subject with significant retinal thickening, who was diagnosed with Frisen grade 1 optic disc edema (right eye), also displayed the largest bilateral increases in ONS area in our cohort, although this increase is relatively small compared to clinical populations with increased ICP^19^. Furthermore, it has been reported that most astronauts develop some degree of retinal thickening based on OCT imaging even if not diagnosed with optic disc edema using ophthalmic imaging. As the ONS is known to be sensitive to changes in CSF pressure^20,21^, these findings suggest that intraorbital CSF pressure may be mildly elevated in astronauts with clinically meaningful optic disc edema. Elevated postflight ICP, measured via lumbar puncture opening pressure, has been reported in astronauts with optic disc edema^3,22^. However, in these previous assessments, opening pressures were not measured using a standardized protocol (e.g., controlling for body position, time after landing, or days from lumbar puncture measure to day of MRI exam), nor were preflight opening pressures available for direct comparison. We also found that intersubject differences in ONS cross-sectional area were much larger than intra-subject differences across time points, demonstrating the necessity for individual baseline measures and an indirect indication of methodological reliability that complements our phantom study. Kramer et al.^6^ subjectively observed a positive relationship between the degree of ONS kinking and ONS diameter. However, in our study, the degree of ON deviation lacked correlation with ONS cross-sectional area (*R*^2^ = 0.22). The trabeculae, pillars, and septa of the subarachnoid space in a region of ONS kinking could compress and compartmentalize CSF pressure along the ON axis^6^ and ultimately lead to the distension of the bulbar region of the ONS that has been previously observed in other astronaut studies^3,6,15,22–24^. As ONS distension and increased kinking of the ONS post flight were not observed in the present study, the data suggest compartmentalization of CSF did not occur.

Previous studies report conflicting results regarding spaceflight-associated changes to the ONS, but were limited to relatively small sample sizes and lacked repeated measures conducted over multiple time points. Mader et al.^3,22,23^ reported ONS distension by MRI in multiple case reports; however, ONS distension was either subjectively observed rather than quantified or the sample size was very low. One case report noted a maximum ONS diameter increase of 0.2 mm preflight to post flight^22^. In another study by Sirek et al.^24^, ultrasound was used to measure ON and ONS diameter before, during, and after spaceflight in 13 astronauts. An average ONS diameter increase of 0.91 mm was reported from pre- to in-flight (*P* = 0.01) and remained distended post flight^24^. However, this study only reported the average results obtained from different astronaut cohorts at each time point. In a study by Kramer et al.^6^, ON and ONS diameters were measured in 27 astronauts (8 of which completed a second mission allowing for control data) with MRI. For the eight astronauts with control scans, there was no significant difference in ONS diameter post flight measured 4 mm posterior to the globe (6.65 ± 1.42 to 6.66 ± 1.72 mm, *P* = 0.97)^6^. In the present study, ONS cross-sectional areas were quantified with MRI in 2D, based on an automated segmentation algorithm. Assuming a circular cross-section, ONS diameters in our study cohort (5.98 ± 0.96 mm preflight and 5.96 ± 0.76 mm post flight at R + 1) (Table 2) exhibited comparable values to those reported by Kramer et al.^6^. Interestingly, Kramer et al.^6^ found that 4 of the 27 astronauts with ON kinking showed significantly increased ONS diameters, with ONS diameter measurements of 7.5 ± 1.1 mm (*P* = 0.03). We, however, observed little change in ONS deviation as a measure of ON kinking for any of the astronauts pre- vs. post flight (Fig. 2c).

On average, ON cross-sectional area tended to decrease in our cohort immediately post flight (at R + 1; Fig. 2b). However, only the R + 30 time point showed a statistically significant reduction in ON cross-sectional area (*P* = 0.04). The R + 30 average change from preflight was −0.89 mm^2^ (95% CI: −1.76 to −0.02), a value relatively large compared to its estimated reproducibility of 0.39 mm^2^ (Table 1) using the phantom model. Although increased CSF pressure surrounding the ON can lead to axoplasmic flow stasis and optic disc edema, as seen in IIH patients, it is unclear how these changes affect the ON morphology^17,25–28^. The exact reason for ON area reduction in our astronaut cohort is unclear and should be confirmed in a larger astronaut cohort. It is also possible that changes in the ON vasculature play a role. A study by Kramer et al.^6^ observed a central ON area with T2-weighted MRI hyperintensity in 26 out of 27 astronauts post flight using a T2-weighted 3D fast spin-echo MRI sequence. In that study, a subset of eight astronauts underwent a repeat study after an additional short-term mission (39 ± 70 days) in space, after which ONS diameter was unchanged. For the remaining astronauts, ONS diameter was measured post flight, but images were not available for comparison to preflight values. In our study, intersubject differences were greater than intra-subject variation and these differences persisted across recovery time points, indicating consistency of the automated ON segmentation method. One outlier showed a large increase in ON area at R + 1 and, notably, this same subject had the largest decrease in ONS area. In addition, the three subjects with an increase in ON area had a concomitant decrease in ONS area at R + 1.

We found no significant change in ON deviation, a measure of ON kinking, after long-duration spaceflight (Fig. 2a). We quantified deviation as the maximum orthogonal distance from the curved ON path to a straight-line path connecting a point 20 mm along the ON to the ON head. We chose to measure the maximum orthogonal distance, as it gives an intuitive measure of ON deviation from a straight path. Similar to the situations with ON and ONS cross-sectional areas, the intersubject differences tended to be larger than intra-subject differences, suggesting reliability of the method. Previous researchers have quantified ON tortuosity as a ratio of ON curved path length to the Euclidean length in healthy adults as well as patients with glaucoma^15,29^.

ON tortuosity has been subjectively reported to occur in astronauts post flight^6,22^. However, a limitation of previous studies is that no preflight comparisons were made; thus, to date, it was not clear whether ON tortuosity developed during spaceflight or if it was already present at baseline. We applied a robust method using multiplanar reconstruction visualization to select the ON centerline. In addition, we conducted an inter- and intra-operator reliability study to help understand the reliability of our ON deviation measurements. Although there were changes in ON deviation greater than the reliability bounds, the observed changes were relatively small on average.

A single subject in our study cohort (subject 2, Figs. 1b and 3c) was diagnosed with grade 1 optic disc edema (i.e., SANS) via ophthalmic imaging. Interestingly, this subject had the largest increase in ONS area immediately post flight (R + 1) and also displayed the largest observed ONS cross-sectional areas both pre- and post flight. It is possible that in-flight ONS distention subsided after landing and before the first postflight scan, or that ICP was elevated before launch and had already saturated the ONS’s compensatory threshold^30^. Previously, Kramer et al.^6^ found a geometrically larger ONS in subjects with globe flattening, another sign of SANS. The subject with grade 1 optic disc edema in our cohort did not exhibit kinks in the ON or ONS and had one of the smallest values observed for ON deviation in this study cohort.

Studies investigating SANS with a spaceflight analog, head-down tilt, have reported ONS distension likely due to elevated ICP in the head-down tilt position^31^. In the present study, we did not observe an increase in ONS cross-sectional area after spaceflight in most individuals, which may indicate ICP is not pathologically elevated in space. Recent studies of a similar astronaut cohort found evidence of ICP elevation in some subjects post flight in the form of pituitary gland deformation and retinal thickening (i.e., optic disc edema)^2^. Alternatively, our findings may indicate that the ONS area decreased immediately upon re-entry into Earth’s gravitational environment, and that any ONS changes that occurred during spaceflight were no longer present by the first MRI scan. The average time to the first postflight MRI scan was 4 ± 2 days after return to Earth and retinal thickness has been shown to decrease at just 3 days post spaceflight compared to in-flight measures^2^.

Background tissue intensities of the fatty tissues surrounding the ON and ONS were used to define MR image offset values on a scan-to-scan basis. If changes occurred to the fatty tissue due to spaceflight, these offset values may have changed. However, when comparing mean pre- and postflight background pixel intensities, these tissues had nearly zero change in pixel intensity, with an average pixel intensity change of only −1.14 ± 5.71 (*P* = 0.14) as compared to an encoding range from 0 to 4095. Other studies utilize histogram mapping techniques to compare MRIs, but these studies suffer from a lack of biological interpretability of the normalized units^32^.

Manual selection of the ON head as a reference point for the purpose of locating the ON position 3 mm posterior to the ONH inherently introduces error. However, we considered this error to be acceptable, as MRI and point cloud registration would be limited by the field of view (FOV) of the scan and movement of the ON. This decision also was impacted by intra-subject variability in the number of assessable slices.

ON deviation assessment utilized T1-weighted MRIs rather than T2-weighted. Although the ON and ONS are generally more differentiable within T2-weighted MRIs, the majority of the nerve becomes indistinguishable 6 mm posterior to the optic globe in such images, whereas T1-weighted scans consistently produced clear images at least 20 mm posterior to the optic globe. To accomplish deviation assessment, only the ON centerline is needed. However, a severe ONS kink could influence the selection of the ON centerline. As we did not observe any major kinks in any nerves, we chose to use the T1-weighted MRIs, which allowed us to analyze a longer region of the ON.

The limited number of long-duration spaceflight astronauts is the primary limitation of this study. Furthermore, although some subjects demonstrate meaningful changes in total retinal thickness, only one subject in this study was diagnosed with Frisen grade 1 optic disc edema. Therefore, it will be critical to evaluate additional astronauts with substantial optic disc edema. Lastly, it was assumed that the number of days of long-duration spaceflight exposure would have a linear effect on ONS cross-sectional area change. However, ONS may have hysteresis such that exposure beyond a certain point may result in failure of the sheath to regress to normal and also change its response to subsequent pressure changes.

Quantitative MRI-based assessment of the ON and ONS before and after spaceflight could help our understanding of SANS and the potential role of ICP. Here we report no change in ON deviation, ON area or ONS areas after immediately post flight, suggesting that on average, CSF pressure within the bulbar subarachnoid space is not pathologically elevated after spaceflight at this time point. While one subject was diagnosed with optic disc edema via ophthalmic imaging and showed an increase in ONS area post flight, most astronauts present with some degree of retinal thickening during and after spaceflight^2^. Further research is warranted to quantitatively assess ON parameters in a larger astronaut cohort.

## Methods

### Data collection

MRI data collection for this study was approved by the NASA and University of Idaho institutional review boards and complied with all relevant ethical regulations for work with human participants and written informed consent was obtained from all participants. All subjects were de-identified before data transfer to the University of Idaho for analysis, although imaging time points for each subject were known.

### Study design

MRI scans were conducted on both the left and right eyes of 10 astronauts participating in the Ocular Health study on a 3T Siemens Verio scanner (Software ver. syngo MR B19, Munich, Germany). High-resolution, T1-weighted sagittal, and T2-weighted coronal scans were collected to assess ON deviation as well as ON and ONS cross-sectional areas, respectively. MRI scans were collected preflight and used as the baseline reference. Five postflight scans were also collected approximately 3, 30, 90, 180, and 360 days after landing. Nine of the ten astronauts completed the immediate postflight scan ~3 days after landing. Because of scan artifacts, over-influence, or lack of scan completion, some astronauts did not complete one or more of the five return scans.

### ON deviation parameters

ON deviation data were available from all 10 astronauts, each supplying 4–12 observations (average 9.2) in total per eye, before and after flight for our multilevel statistical analysis. Astronauts were instructed to fixate on a target straight ahead during MR imaging, as gaze may impact ON deviation measurements. MR image artifacts were present in two data sets and did not allow for ON deviation assessment. Two observations were eliminated from our analysis because of over-influence (1 preflight OD observation and 1R + 30 OD observation, both from the same astronaut), after which our statistical model residuals and assumption testing passed.

**Fig. 4:**
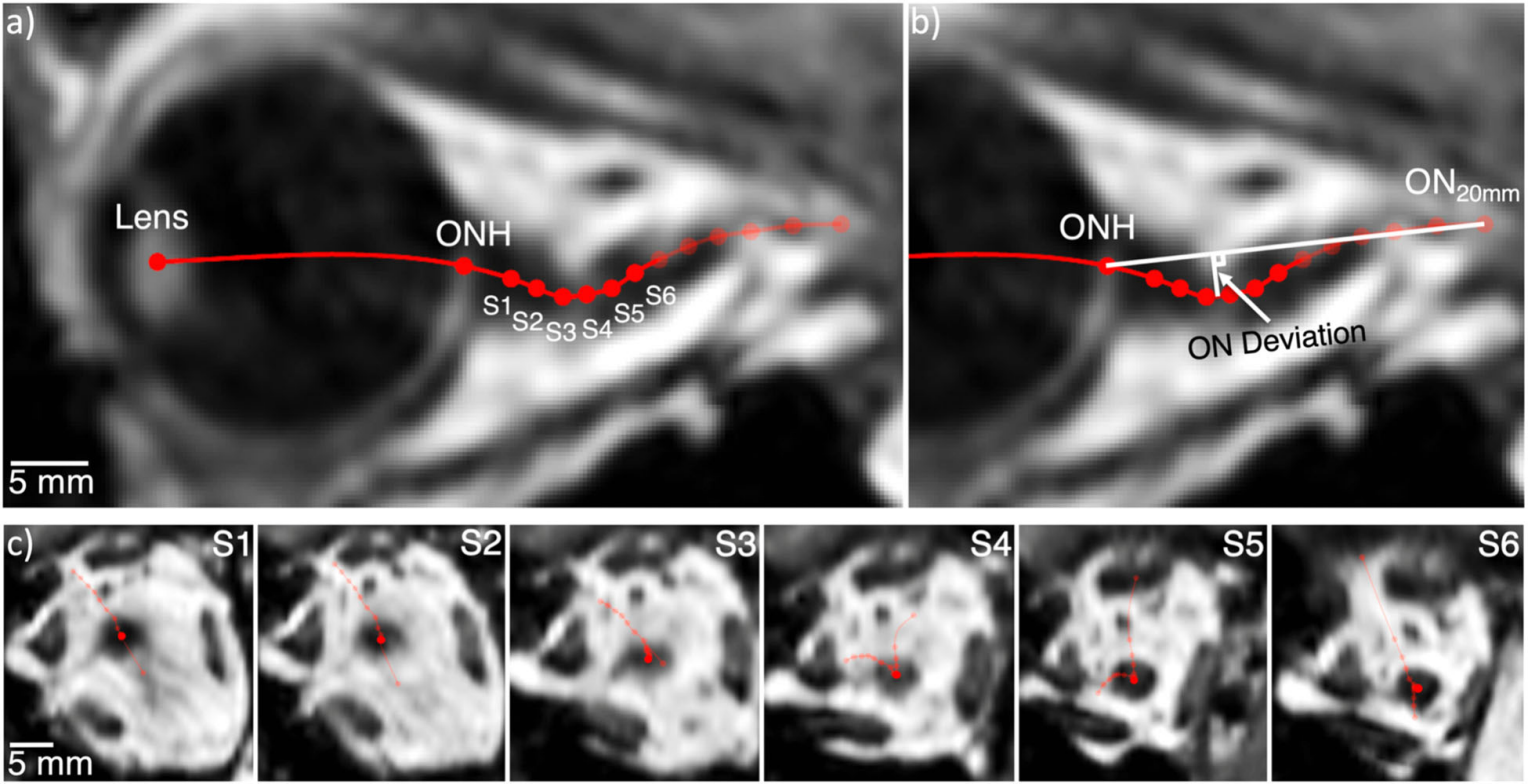
Methods for computing ON deviation. **a** Visualization of the ON centerline path (red), based on manual selection of points (lens center, optic nerve head, and six points on the optic nerve [S1–S6]). **b** Visualization of the ON deviation (white arrow), measured as the distance between interpolated points along the ON trajectory (red) and the corresponding straight line from optic nerve head to final point along ON centerline path (white). **c** 3D multiplanar reconstruction showing coronal view of each location (S1–S6) with ON centerline paths (red).

To quantify the degree of ON “kinking” (Fig. 1), the following methods were applied to obtain ON deviation for each astronaut pre- and post flight. A volume of T1-weighted images was collected (Fig. 4) with 900 μm slice thickness and spacing, 488 μm in-plane isotropic pixel size (FOV 512 × 512), repetition time TR = 1900, echo time TE = 2.32, and 9° flip-angle. Curved ON trajectories were manually generated using the 3D curved multiplanar reconstruction tool within the OsirixMD DICOM viewer (version 8.0.1, Pixmeo, Geneva, Switzerland). Points along the ON centerline trajectory were selected with ~1–2 mm spacing and exported as lists of comma-separated (X, Y, Z)-coordinates (Fig. 4a, c). An upsampled spline curve was fit to the points and truncated at a centerline trajectory length of 20 mm posterior to the ONH using MATLAB (Ver. 2015a, Mathworks, Natick, MA). A trajectory length of 20 mm was used because this length covered the region of ON kinking previously reported to be present in some astronauts^6^. The lens center and ON head locations in 3D space were also identified based on multiplanar visual inspection. ON deviation was determined based on the maximum orthogonal distance between the curved ON centerline trajectory and a straight line connecting the ON head and the point on the ON trajectory 20 mm posterior to the ON head (Fig. 4b).

### ON and ONS geometric parameters

ON cross-sectional areas were analyzed in all 10 astronauts, each supplying 2–11 observations (average 6.5) in total per eye, before and after flight for our multilevel statistical analysis, with some missing data because of MR image artifacts or missing scans (pre- or postflight high-resolution, T2-weighted, coronal MRI data not collected). Two ON area observations were again eliminated due to over-influence and skewed residuals (both from the same astronaut’s OD eye, 1 immediately post flight, the other at R + 90). ONS cross-sectional areas were analyzed in all 10 astronauts, each supplying 2–11 observations (average 6.7) in total per eye, before and after flight for our multilevel statistical analysis.

To assess ONS distension, 3D geometries of the ON and ONS were quantified using the following semi-automated method. High-resolution, T2-weighted, coronal MRI scans were collected (Fig. 3a) with 600 μm slice thickness and spacing, 253 μm in-plane isotropic pixel size (FOV 256 × 256), repetition time TR = 820, echo time TE = 118, and 170° flip-angle. To extract ON and ONS contours, we used the following adaptive thresholding process. First, average background pixel intensity for each scan was computed, with the intensity ranging from 0 to 4095 (12 bit image), excluding the influence of potential volumetric changes in CSF. The background was selected with a slice-by-slice mask relative to the peak frequency in the pixel intensity histogram. The average and SD of background tissue pixel intensity change was −1.14 ± 5.71 (*P* = 0.14). A threshold was then chosen by adding the difference between the average intensity across all scans and the current scan to a common value. MRI slices were cubically upsampled and contoured in MATLAB with the computed threshold (Fig. 3b). ON and ONS contours were automatically selected using a point count filter and isoperimetric difference quotient (roundness measure). The ON head location was manually specified for each case based on multiplanar reconstruction. Linear interpolation between contours (600 μm slice spacing) was applied to obtain a single contour located 3 mm posterior to the ON head along the nerve trajectory. Contours at varying distances from the ON head also could be generated providing a 3D representation of the ON and ONS (Fig. 3c). ON and ONS cross-sectional areas 3 mm posterior to the ON head were then computed.

### Reliability assessment

The inter-operator reliability of the ON deviation parameter was quantified using four trained expert operators masked to each other’s ratings. Each operator manually measured all parameters on three representative MRI data sets on a single day and repeated these measurements on three separate occasions separated by at least a 3-day interval to reduce possible influence of memory on trajectory point selections. Intra-operator reliability was quantified based on five measurements recorded by a single operator, with at least 3-day intervals, for the same three MRI data sets. The average value for each ON deviation parameter was computed for the inter- and intra-operator reliability studies. The reliability of the ON deviation measurement parameter was computed as the mean difference of the individual cases from the average value and the standard deviation of the mean differences. As there is no gold standard for this measurement, the mean value of all reliability measurements was assumed to be the true value.

ON and ONS cross-sectional area reliability was quantified by conducting an MRI phantom study. A high-resolution phantom model was created that contained three idealized optic subarachnoid space geometries and three subject-specific geometries, each with a 2 cm axial length. Idealized geometries had an ONS diameter of 7 mm at one end of the model, tapering to 5 mm. An ON with a diameter of 3.35 mm was located concentrically in ONS. The subject-specific geometry was created based on a segmentation of a representative astronaut optic subarachnoid space produced by the above methods. The phantom model was printed in WaterShed XC material (Koninklijke DSM N.V., Heerlen, The Netherlands) by high-resolution stereolithography printing technology with a 50 μm layer thickness and 15 μm in-plane resolution. The phantom was scanned twice on the same 3T MRI machine used for astronauts in this study. MRI were processed using an identical procedure used to assess the ON and ONS cross-sectional area in the astronauts.

### Statistical analyses

Statistical analyses were conducted using Stata/SE (v 16.0), setting two-tailed alpha to reject the null hypothesis at 0.05, with an emphasis on characterizing the observed effects in addition to reporting statistical significance. Our experimental design was a mixed-factorial with repeated observations nested within the astronaut (left, right eye) and over time (preflight and several postlanding time points). All of our outcomes were continuously scaled and were analyzed using Gaussian-based maximum likelihood mixed-effects modeling including two random Y-intercepts for the nesting of left and right eye measurements within time period and to accommodate for the repeated measures over time. We included a fixed-effects covariate parameter to adjust for astronauts’ prior microgravity exposure (i.e., the number of days flown prior) and fixed-effects β-coefficients comparing each postflight observation to preflight. Given the large number of planned pairwise comparisons (preflight vs. each postflight), we employed and report Bonferroni adjustments for the inflated risk of Type I errors. Model residuals were closely examined for overly influential outlier observations and normality. Individual observations producing standardized residual greater than 2 SE’s above or below the mean, and resulting in a significantly skewed distribution, were deemed overly influential and were eliminated from the analysis and are reported in the results accordingly.

### Reporting summary

Further information on research design is available in the Nature Research Reporting Summary linked to this article.

## Supplementary information

Reporting Summary

## Data Availability

The data sets generated during and/or analyzed during the current study are available from the Research Data Repository (LDSA), https://lsda.jsc.nasa.gov/, and can be requested at this site. Requests are only granted following a thorough review process and Johnson Space Center international review board approval in accordance with the Privacy Act of 1974.

## References

[1] Lee AG, Mader TH, Gibson CR, Brunstetter TJ, Tarver WJ (2018). Space flight-associated neuro-ocular syndrome (SANS). Eye.

[2] Macias BR (2020). Association of long-duration spaceflight-induced anterior and posterior ocular structure changes and their recovery. JAMA Opthalmol.

[3] Mader TH (2011). Optic disc edema, globe flattening, choroidal folds, and hyperopic shifts observed in astronauts after long-duration space flight. Ophthalmology.

[4] Marshall-Bowman K, Barratt MR, Gibson CR (2013). Ophthalmic changes and increased intracranial pressure associated with long duration spaceflight: An emerging understanding. Acta Astronautica.

[5] Lee AG (2016). Neuro-ophthalmology of space flight. J. Neuroophthalmol..

[6] Kramer LA, Sargsyan AE, Hasan KM, Polk JD, Hamilton DR (2012). Orbital and intracranial effects of microgravity: findings at 3-T MR imaging. Radiology.

[7] Zhang LF, Hargens AR (2018). Spaceflight-induced intracranial hypertension and visual impairment: pathophysiology and countermeasures. Physiol. Rev..

[8] Feola AJ, Nelson ES, Myers J, Ethier CR, Samuels BC (2018). The impact of choroidal swelling on optic nerve head deformation. Invest. Ophthalmol. Vis. Sci..

[9] Feola AJ (2017). Deformation of the lamina cribrosa and optic nerve due to changes in cerebrospinal fluid pressure. Invest. Ophthalmol. Vis. Sci..

[10] Raykin J (2017). Characterization of the mechanical behavior of the optic nerve sheath and its role in spaceflight-induced ophthalmic changes. Biomech. Model Mechanobiol..

[11] Feola AJ (2016). Finite element modeling of factors influencing optic nerve head deformation due to intracranial pressure. Invest. Ophthalmol. Vis. Sci..

[12] Miyati T (2007). Noninvasive MRI assessment of intracranial compliance in idiopathic normal pressure hydrocephalus. J. Magn. Reson. Imaging.

[13] Armstrong GT (2007). Defining optic nerve tortuosity. AJNR Am. J. Neuroradiol..

[14] Campen CJ, Gutmann DH (2018). Optic pathway gliomas in neurofibromatosis type 1. J. Child Neurol..

[15] Scott, R., Tarver, W., Brunstetter, T. & Urquieta, E. Optic nerve tortuosity on earth and in space. *Aerosp. Hum. Perform.* **91**, 7 (2020). 10.3357/AMHP.5406.2020 31980047

[16] Jensen RH, Radojicic A, Yri H (2016). The diagnosis and management of idiopathic intracranial hypertension and the associated headache. Ther. Adv. Neurol. Disord..

[17] Rigi M, Almarzouqi SJ, Morgan ML, Lee AG (2015). Papilledema: epidemiology, etiology, and clinical management. Eye Brain.

[18] Thurtell MJ, Wall M (2013). Idiopathic intracranial hypertension (pseudotumor cerebri): recognition, treatment, and ongoing management. Curr. Treat. Options Neurol..

[19] Geeraerts T (2008). Use of T2-weighted magnetic resonance imaging of the optic nerve sheath to detect raised intracranial pressure. Crit. Care.

[20] Xie X (2013). Noninvasive intracranial pressure estimation by orbital subarachnoid space measurement: the Beijing Intracranial and Intraocular Pressure (iCOP) study. Crit. Care.

[21] Hansen HC, Helmke K (1997). Validation of the optic nerve sheath response to changing cerebrospinal fluid pressure: ultrasound findings during intrathecal infusion tests. J. Neurosurg..

[22] Mader TH (2013). Optic disc edema in an astronaut after repeat long-duration space flight. J. Neuroophthalmol..

[23] Mader TH (2017). Persistent asymmetric optic disc swelling after long-duration space flight: implications for pathogenesis. J. Neuroophthalmol..

[24] Sirek AS (2014). Doppler ultrasound of the central retinal artery in microgravity. Aviat. Space Environ. Med..

[25] Lochner P (2016). Feasibility and usefulness of ultrasonography in idiopathic intracranial hypertension or secondary intracranial hypertension. BMC Neurol..

[26] Bauerle J, Nedelmann M (2011). Sonographic assessment of the optic nerve sheath in idiopathic intracranial hypertension. J. Neurol..

[27] Hoffmann J (2014). Volumetric assessment of optic nerve sheath and hypophysis in idiopathic intracranial hypertension. AJNR Am. J. Neuroradiol..

[28] Hoffmann J (2019). The effect of CSF drain on the optic nerve in idiopathic intracranial hypertension. J. Headache Pain..

[29] Wang, X. et al. Optic nerve tortuosity and globe proptosis in normal and glaucoma subjects. *J. Glaucoma.*10.1097/IJG.0000000000001270 (2019). 10.1097/IJG.0000000000001270 31045951

[30] Wostyn P, De Deyn PP (2017). Optic nerve sheath distention as a protective mechanism against the visual impairment and intracranial pressure syndrome in astronauts.. Invest. Ophthalmol. Vis. Sci..

[31] Marshall-Goebel K (2017). Lower body negative pressure reduces optic nerve sheath diameter during head-down tilt. J. Appl. Physiol..

[32] Shinohara RT (2014). Statistical normalization techniques for magnetic resonance imaging. Neuroimage Clin..

