# Supplementary material for: Quantitative magnetic resonance image assessment of the optic nerve and surrounding sheath after spaceflight
Source: NPJ Microgravity. 2020 Oct 8;6:30. doi: 10.1038/s41526-020-00119-3 (PMC7545196; doi:10.1038/s41526-020-00119-3)
Supplement: Supplementary file 1 — Reporting Summary [file 41526_2020_119_MOESM1_ESM.pdf]

nature research

Corresponding author(s): Bryn Martin

Last updated by author(s): Aug 27, 2020

Reporting Summary

Nature Research wishes to improve the reproducibility of the work that we publish. This form provides structure for consistency and transparency in reporting. For further information on Nature Research policies, see Authors & Referees and the Editorial Policy Checklist.

Statistics

For all statistical analyses, confirm that the following items are present in the figure legend, table legend, main text, or Methods section.

n/a	Confirmed	
☐	☒	The exact sample size (*n*) for each experimental group/condition, given as a discrete number and unit of measurement
☐	☒	A statement on whether measurements were taken from distinct samples or whether the same sample was measured repeatedly
☐	☒	The statistical test(s) used AND whether they are one- or two-sided<br>*Only common tests should be described solely by name; describe more complex techniques in the Methods section.*
☐	☒	A description of all covariates tested
☐	☒	A description of any assumptions or corrections, such as tests of normality and adjustment for multiple comparisons
☐	☒	A full description of the statistical parameters including central tendency (e.g. means) or other basic estimates (e.g. regression coefficient) AND variation (e.g. standard deviation) or associated estimates of uncertainty (e.g. confidence intervals)
☐	☒	For null hypothesis testing, the test statistic (e.g. *F*, *t*, *r*) with confidence intervals, effect sizes, degrees of freedom and *P* value noted<br>*Give P values as exact values whenever suitable.*
☒	☐	For Bayesian analysis, information on the choice of priors and Markov chain Monte Carlo settings
☐	☒	For hierarchical and complex designs, identification of the appropriate level for tests and full reporting of outcomes
☐	☒	Estimates of effect sizes (e.g. Cohen's *d*, Pearson's *r*), indicating how they were calculated

Our web collection on statistics for biologists contains articles on many of the points above.

Software and code

Policy information about availability of computer code

Data collection	Siemens 3T MRI.
Data analysis	Image post-processing in OsiriX MD (version 8.0.1, Pixmeo, Geneva, Switzerland), MATLAB (Ver. 2015a, Mathworks, Natick, MA), and Stata/SE (v 16.0) software.

For manuscripts utilizing custom algorithms or software that are central to the research but not yet described in published literature, software must be made available to editors/reviewers. We strongly encourage code deposition in a community repository (e.g. GitHub). See the Nature Research guidelines for submitting code & software for further information.

Data

Policy information about availability of data

All manuscripts must include a data availability statement. This statement should provide the following information, where applicable:

- Accession codes, unique identifiers, or web links for publicly available datasets
- A list of figures that have associated raw data
- A description of any restrictions on data availability

Data are available on request due to privacy or other restrictions

Field-specific reporting

Please select the one below that is the best fit for your research. If you are not sure, read the appropriate sections before making your selection.

☒ Life sciences ☐ Behavioural & social sciences ☐ Ecological, evolutionary & environmental sciences

October 2018

Life sciences study design

All studies must disclose on these points even when the disclosure is negative.

Sample size	This study includes analysis of MRI data collected for 10 long-duration spaceflight astronauts (>6 months on the international space station). This was the maximum number of data sets that were possible to analyze for the study at the time of publication.
Data exclusions	Nine of the 10 astronauts completed the immediate postflight scan ~3 days after landing. Because of scan artifacts, overinfluence, or lack of scan completion, some astronauts did not complete 1 or more of the 5 return scans.
Replication	The inter-operator reliability of the optic nerve (ON) deviation parameter was quantified using 4 trained expert operators masked to each other's ratings. Each operator manually measured all parameters on 3 representative MRI data sets on a single day. Each operator repeated these measurements on 3 separate occasions separated by at least a 3-day interval to reduce possible influence of memory on trajectory point selections. Intra-operator reliability was quantified based on 5 measurements recorded by a single operator, with at least 3-day intervals, for the same 3 MRI data sets. The average value for each ON deviation parameter was computed for the inter and intra-operator reliability studies. The reliability of the ON deviation measurement parameter was computed as the mean difference of the individual cases from the average value and the standard deviation of the mean differences. Because there is no gold standard for this measurement, the mean value of all reliability measurement was assumed to be the true value.<br>ON and optic nerve sheath (ONS) cross-sectional area reliability was quantified by conducting an MRI phantom study. A high-resolution phantom model was created that contained 3 idealized optic subarachnoid space geometries and 3 subject-specific geometries, each with a 2 cm axial length. Idealized geometries had an ONS diameter of 7 mm at one end of the model, tapering to 5 mm. An ON with a diameter of 3.35 mm was located concentrically in ONS. The subject-specific geometry was created based on a segmentation of a representative astronaut optic subarachnoid space produced by the above methods. The phantom model was printed in WaterShed XC material (Koninklijke DSM N.V., Heerlen, Netherlands) by high-resolution SLA printing technology with a 50 µm layer thickness and 15 µm in-plane resolution. The phantom was scanned twice on the same 3T MRI machine used for astronauts in this study. MRI images were processed using an identical procedure used to assess the ON and ONS cross-sectional area in the astronauts.<br>Methods were found to be reproducible for all parameters analyzed.
Randomization	It was not possible to randomize any intervention for the astronauts enrolled in our study.
Blinding	Researchers were blinded to the study group in terms of which astronauts developed spaceflight-associated neuro-ocular syndrome (SANS).

Reporting for specific materials, systems and methods

We require information from authors about some types of materials, experimental systems and methods used in many studies. Here, indicate whether each material, system or method listed is relevant to your study. If you are not sure if a list item applies to your research, read the appropriate section before selecting a response.

Materials & experimental systems

n/a	Involved in the study	
☒	☐	Antibodies
☒	☐	Eukaryotic cell lines
☒	☐	Palaeontology
☒	☐	Animals and other organisms
☐	☒	Human research participants
☒	☐	Clinical data

Methods

n/a	Involved in the study	
☒	☐	ChIP-seq
☒	☐	Flow cytometry
☐	☒	MRI-based neuroimaging

Human research participants

Policy information about studies involving human research participants

Population characteristics	Noninvasive MRI data were collected in 10 astronauts preflight (508 ± 230 days before launch) and at 5 recovery time-points after return (R+) to Earth: R+1/3 (4 ± 2 days), R+30 (31 ± 5 days), R+90 (101 ± 16 days), R+180 (188 ± 15 days), and R+360 (355 ± 14 days). The means ± standard deviations of height, weight, and flight duration for this cohort were 1.8 ± 0.1 m, 76 ± 9 kg, and 167 ± 17 days. Only one subject in our study cohort was diagnosed with grade 1 optic disc edema via fundus imaging (i.e., SANS).
Recruitment	Astronauts in our study were recruited by standard NASA protocols under IRB regulations and had informed consent.
Ethics oversight	NASA IRB

Note that full information on the approval of the study protocol must also be provided in the manuscript.

Magnetic resonance imaging

Experimental design

Design type: Pre- versus post-flight MRI collected for all astronauts. See exact time points in text above.

Design specifications: See design specifications in population characteristics text above.

Behavioral performance measures: None.

Acquisition

Imaging type(s): Structural

Field strength: 3T

Sequence & imaging parameters: To quantify the degree of ON “kinking”, the following methods were applied to obtain ON deviation for each astronaut pre- and postflight. A volume of T1-weighted images was collected with 900 µm slice thickness and spacing, 488 µm in-plane isotropic pixel size (FOV 512 x 512), repetition time TR=1900, echo time TE = 2.32, and 9? flip-angle.

To assess ONS distension, 3D geometries of the ON and ONS were quantified using the following semi-automated method. High-resolution T2-weighted coronal MRI scans were collected with 600 µm slice thickness and spacing, 253 µm in-plane isotropic pixel size (FOV 256 x 256), repetition time TR = 820, echo time TE = 118, and 170? flip-angle.

Area of acquisition: Whole brain.

Diffusion MRI: ☐ Used ☐ Not used

Preprocessing

Preprocessing software: None

Normalization: To extract ON and ONS contours, we used the following adaptive thresholding process. First, average background pixel intensity for each scan was computed, with the intensity ranging from 0 to 4095 (12-bit image), excluding the influence of potential volumetric changes in CSF. The background was selected with a slice-by-slice mask relative to the peak frequency in the pixel intensity histogram. The average and standard deviation of background tissue pixel intensity change was -1.14 ± 5.71 (P = 0.14). A threshold was then chosen by adding the difference between the average intensity across all scans and the current scan to a common value.

Normalization template: N/A

Noise and artifact removal: N/A

Volume censoring: N/A

Statistical modeling & inference

Model type and settings: Statistical analyses were conducted using Stata/SE (v 16.0), setting 2-tailed alpha to reject the null hypothesis at 0.05, with an emphasis on characterizing the observed effects in addition to reporting statistical significance. Our experimental design was a mixed-factorial, with repeated observations nested within astronaut (left, right eye) and over time (Preflight and several post-landing time points). All of our outcomes were continuously scaled and were analyzed using Gaussian-based maximum likelihood mixed-effects modeling including 2 random Y-intercepts for the nesting of left and right eye measurements within time period and to accommodate for the repeated-measures over time. We included a fixed-effects covariate parameter to adjust for astronauts’ prior microgravity exposure (i.e. the number of days flown prior), and fixed effects beta coefficients comparing each postflight observation to preflight. Given the large number of planned pairwise comparisons (preflight versus each postflight), we employed and report Bonferroni adjustments for the inflated risk of Type I errors. Model residuals were closely examined for overly influential outlier observations and normality. Individual observations producing standardized residual greater than 2 SE’s above or below the mean, and resulting in a significantly skewed distribution, were deemed overly influential and were eliminated from the analysis and are reported in the results accordingly.

Effect(s) tested: See text above for model type and settings.

Specify type of analysis: ☐ Whole brain ☒ ROI-based ☐ Both

Anatomical location(s): Visual inspection to determine location of optic nerve and nerve sheath.

Statistic type for inference
(See Eklund et al. 2016)

Voxel-wise. A threshold was then chosen by adding the difference between the average intensity across all scans and the current scan to a common value. MRI slices were cubically up-sampled and contoured in MATLAB with the computed threshold. ON and ONS contours were automatically selected using a point count filter and isoperimetric difference quotient (roundness measure). The optic nerve head location was manually specified for each case based on multiplanar reconstruction. Linear interpolation between contours (600 µm slice spacing) was applied to obtain a single contour located 3 mm posterior to the optic nerve head along the nerve trajectory. Contours at varying distances from the optic nerve head also could be generated providing a 3D representation of the ON and ONS. ON and ONS cross-sectional areas 3 mm posterior to the optic nerve head were then computed.

Correction	See statistics above.

Models & analysis

n/a	Involved in the study
☒	☐ Functional and/or effective connectivity
☒	☐ Graph analysis
☒	☐ Multivariate modeling or predictive analysis
